# The Effects of Compliance with Nutritional Counselling on Body Composition Parameters in Head and Neck Cancer Patients under Radiotherapy

**DOI:** 10.1155/2017/8631945

**Published:** 2017-01-01

**Authors:** D. Hopanci Bicakli, O. Ozkaya Akagunduz, R. Meseri Dalak, M. Esassolak, R. Uslu, M. Uyar

**Affiliations:** ^1^Department of Medical Oncology, Ege University School of Medicine, Bornova, 35100 Izmir, Turkey; ^2^Department of Radiation Oncology, Ege University School of Medicine, Bornova, 35100 Izmir, Turkey; ^3^Department of Nutrition and Dietetics, Ege University Izmir Ataturk School of Health, Bornova, 35100 Izmir, Turkey; ^4^Department of Anesthesiology and Intensive Care, Ege University School of Medicine, Bornova, 35100 Izmir, Turkey

## Abstract

*Background*. Radiotherapy (RT) has been associated with increased risk of malnutrition in cancer patients, particularly in those with head and neck cancer (HNC). The aim of this prospective study was to evaluate the effects of compliance of patients with individual dietary counselling on body composition parameters in HNC patients under RT.* Material and Methods*. Sixty-nine consecutive patients (mean age: 61.0 ± 13.8) were prospectively followed. Bioelectrical impedance analysis (BIA) was performed to determine body composition parameters before, in the middle of, and at the end of RT. All patients received nutritional counselling and majority of them (94.6%) received oral nutritional supplement (ONS) during RT or chemoradiotherapy. If a patient consumed ≥75% of the recommended energy and protein intake via ONS and regular food, he/she was considered to be “compliant” (*n* = 18), while those who failed to meet this criteria were considered to be “noncompliant” (*n* = 30).* Results*. Body mass index, weight, fat percentage, fat mass, fat free mass, and muscle mass did not decrease significantly over time in compliant patients, but in noncompliant patients, all of these indices decreased significantly from baseline compared to the end of treatment (*p* < 0.001). Hand grip strength did not differ significantly between the two groups at baseline and over time in each group. When retrospectively evaluated, heavy mucositis was less commonly observed in compliant than noncompliant patients (11.1% versus 88.9%, resp.) (*p* < 0.009).* Conclusion*. We conclude that body composition parameters were better in head and neck cancer patients considered as compliant with nutritional counselling than noncompliant ones during RT period.

## 1. Introduction

Head and neck cancer (HNC) refers to tumours of all anatomical structures that extend from the nasopharynx to the cervical oesophagus. Environmental factors such as smoking and alcohol consumption may contribute to development of HNC [1]. Cancer treatments are associated with increased risk of nutritional deterioration. Radiotherapy (RT) and/or chemoradiotherapy (CRT) induce symptoms such as mucositis, chewing and swallowing difficulties, anorexia, and xerostomia [2, 3]. In studies including only HNC patients, it is stated that, along with other problems related to anatomical localization of the tumour, these symptoms may lead to weight loss in around 50% of cases under RT [2, 3] and in as much as 80% of cases under CRT with likelihood of nausea and vomiting, loss of taste, and loss of appetite caused by the toxicity of the CRT [4]. The reasons of weight loss in 20–30% of cases who lost are related severe mucositis and dysphagia in a similar patient population [5]. Severe weight loss has been associated with a reduction in physical activity, an increase in treatment related toxicity, a poor response to treatment, and increase in susceptibility to infection due to reduction in immunoresistance. The prevention as well as early diagnosis of malnutrition is of the utmost importance in such patients [6–8]. Tailoring of nutritional support for cancer patients at the beginning of treatment can improve the patient's quality of life by maintaining body weight and helping a range of issues in order to tolerate the treatment [9, 10].

The aim of this prospective study was to evaluate the effect of compliance with individual dietary counselling provided by the dietitian on body composition and anthropometry in HNC patients under RT.

## 2. Material and Methods

### 2.1. Study Population

**Figure 1:**
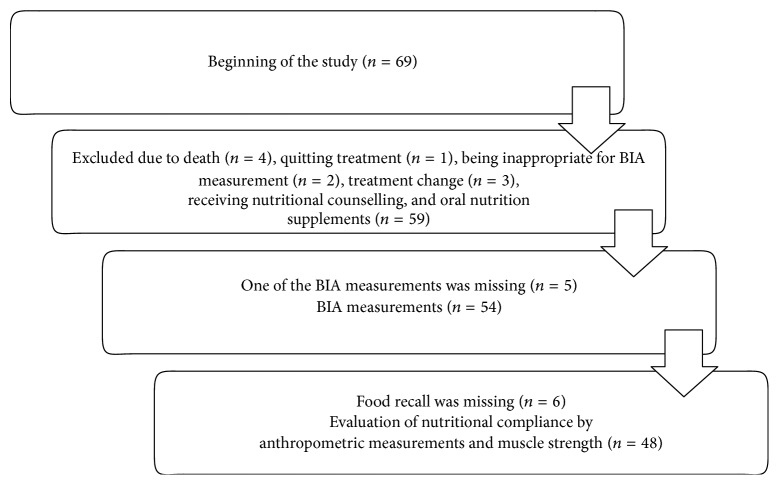
Flowchart of the study.

A total of 69 ambulatory or hospitalized patients with HNC who were referred to the Department of Radiation Oncology for adjuvant or definitive RT with or without CT and received nutritional counselling and oral nutritional supplements (ONS) during RT or CRT were enrolled in this prospective single-center study at Ege University Hospital, Izmir, Turkey. As shown in study flow chart (Figure 1), final study population was composed of 59 patients with exclusion of 10 patients from the study due to death (*n* = 4), treatment withdrawal (*n* = 1), treatment alteration (*n* = 3), and occurrence of a physical condition that might affect study parameters (*n* = 2).

Written informed consent was obtained from each subject following a detailed explanation of the objectives and protocol of the study which was conducted in accordance with the ethical principles stated in the “Declaration of Helsinki” and approved by the Ege University Ethics Committee.

### 2.2. Study Parameters

Data on baseline characteristics (diagnosis, disease stage, type of chemotherapy, and surgery), percent weight loss, and ONS consumption characteristics (type of ONS, regular/irregular consumption, and reasons for irregular consumption) were recorded in each patient. Anthropometrics [body weight (kg), body mass index (BMI; kg/m^2^)] and body composition parameters [% fat, fat mass (kg), fat free mass (kg), and muscle mass (kg)], muscle function (hand grip strength), and severity of mucositis were evaluated according to patient compliance with nutritional intervention.

In 5 out of 59 patients one of the BIA measurements was missing; thus anthropometric measurements were evaluated in 54 patients. Food recall was missing in 6 patients and the association of nutritional compliance with anthropometric measurements and muscle strength was assessed in 48 patients (Figure 1).

Anthropometric and bioelectrical impedance analysis (BIA) measurements were performed at baseline (in the first day of treatment), in the middle of, and at the end of the treatment. Body weight (kg), BMI (kg/m^2^), fat percentage, fat mass (FM), fat-free mass (FFM), and muscle mass (MM) were measured using TANITA (Tanita Body Composition Analyzer SC 330 Japan). Grip strength was measured as identified of muscle strength, by means of a hand dynamometer (Grip-D T.K.K.5401 Japan) in a sitting position, on the nondominant hand, and with the elbow fixed at 90 degrees three consecutive times and the mean was taken for analysis. Mucositis was evaluated according to Radiation Therapy Oncology Group (RTOG) toxicity criteria [11].

### 2.3. RT with or without CRT

Patients were treated with intensity-modulated RT or three-dimensional conformal RT. Patients received radiation doses that ranged from 60 to 70 Gy. The chemotherapeutic regimen included three cycles of neoadjuvant cisplatin (75 mg/m^2^) and docetaxel (75 mg/m^2^), followed by three weekly cisplatin (75 mg/m^2^) concurrently with RT or weekly cisplatin (40 mg/m^2^) with RT or cetuximab (one cycle of neoadjuvant 400 mg/m^2^ followed by 250 mg/m^2^ weekly with RT).

### 2.4. Nutritional Counselling and Oral Nutritional Supplements

All patients received nutritional counselling during RT or CRT. Dietary counselling was provided by the same dietitian before, in the middle of, and at the end of RT with approximately two-week intervals. Regular and nutritional counselling was provided to each patient in accordance with their individual requirements based on the extent of their malnutrition, the prognosis and stage of their illness, and the side effects of treatment. Individually intended sample meal plans, recipe advices, and suggestions in order to minimise the side effects of the tumour and therapy such as mucositis, nausea, or vomiting were provided. Oral hypercaloric nutritional supplements and protein supplements were also provided. When symptoms such as dysphagia, swallowing difficulties, appetite loss, nausea, taste problems, or xerostomia were developed under RT, the individualised dietary modification was implemented to meet patients' needs.

Mean energy and protein intake were calculated from three-day food recall. Each patient was instructed by the dietician on how to fill in the diary. Dietary records were analyzed for energy and protein content using country-specific food composition tables for Turkey [12].

Three-day food recall was taken consequently in the middle of the treatment, when the second anthropometric measurement was conducted. Energy and protein intake per unit weight were calculated for mean energy and protein intake divided by weight obtained from the second measurement. If the patient consumed ≥75% of the recommended energy and protein by taking ONS and regular food, he/she was considered as a “compliant patient” while those who consumed <75% of the recommended energy and protein via ONS or regular food were considered to be “noncompliant patients.” Patients having problems like pain, mucositis, swallowing difficulties, diarrhea, bad taste, and loss of appetite were also considered as noncompliant.

### 2.5. Statistical Analysis

Continuous variables were presented as means ± standard deviation and categorical variables were summarized as percentages. Effect of regular consumption of ONS on total energy and protein intake was determined by chi-square and Mann–Whitney *U* test. Change in anthropometric measurements and body composition (weight, fat free mass, fat mass, fat%, muscle, and BMI) and muscle strength within time in compliant and noncompliant patients were evaluated via Friedman Repeated measurements, whereas the association between mucositis and consumption was determined by chi-square test. Data were analyzed using SPSS version 15.0 software. Significance was defined as *p* < 0.05.

## 3. Results

In total 59 patients were evaluated. Among them, 79.7% were male and the mean age was 61.0 ± 13.8. Patient characteristics are shown in Table 1.

As shown in Table 1, half (49.2%) of the patients had larynx-hypopharynx cancer at various stages. Most of the patients (94.9%) had squamous cell cancer type tumours. Mean RT duration was 43.5 ± 5.6 days. Only three of the patients had diabetes, and one had hypertension.

The percent weight loss of patients at onset of RT (according to their usual weight), the recommended ONS, consumption status, and main reasons for nonconsumption of ONS of the patients are presented in Table 2.

Hypercaloric ONS was recommended for majority of patients (94.6%), while only 55.4% of patients were identified to consume ONS on a regular basis. Gastrointestinal disorders were the main reason for irregular ONS consumption (Table 2).

Three-day food recall could not be received from six patients. Thus daily energy and protein intake were evaluated in 53 patients. Mean daily energy and protein intake were 1518.8 ± 522.5 kCal and 66.1 ± 30.3 grams, respectively. When patients' weight was considered, mean energy and protein intake per unit weight were 21.8 ± 9.3 kCal and 0.95 ± 0.5 grams, respectively. Thirty-two patients (60.4%) had sufficient energy intake and twenty-one patients (39.6%) had sufficient protein intake. Thus, 20 patients (38%) who consumed sufficient (≥75% of their requirements) energy and protein were considered to be “compliant” (data on BIA measurements were not available in 2 patients). Consumption did not meet the criteria of ≥75% of the recommended energy and protein intake in 62% (*n* = 33) of patients, who were therefore considered to be “noncompliant” (data on BIA measurements were not available in 3 patients).

Mean total and per unit weight energy [1977.1 ± 385.3 kcal (29.3 ± 7.1 kcal/kg) versus 1240.9 ± 380.2 kcal (17.2 ± 72 kcal/kg), *p* < 0.001] and protein [97.8 ± 16.6 gr (1.5 g ± 0.4 gr/kg) versus 46.8 ± 17.8 gr (0.6 ± 0.3 gr/kg), *p* < 0.001] intake were significantly higher in compliant than in noncompliant patients. Similarly, regular ONS consumers had significantly higher intake of energy per unit weight (*p* < 0.001) and protein per unit weight (*p* < 0.001). Three patients had nasogastric tube whereas 4 had percutaneous endoscopic gastrostomy (PEG).

Nine patients were hospitalized, 29 patients were staying at home or with relatives, and 11 were staying at a hotel during the treatment period. Compared to those staying in a hotel, those staying at home or with relatives had significantly higher energy intake (*p* = 0.009), while no significant difference was noted with respect to protein intake.

No significant difference was noted between compliant and noncompliant patients at baseline in terms of BMI, weight, fat percentage, fat mass, fat free mass, muscle mass, and hand grip strength. Change in body composition over the time is presented in Table 3.

No significant change was noted in BMI, weight, fat percentage, fat mass, fat free mass, and muscle mass values over the time in compliant patients, whereas in noncompliant patients all these indices decreased significantly from baseline to the end of the treatment (*p* < 0.001).

Hand grip strength at baseline was similar in compliant and noncompliant patients. Also, no significant change was noted in hand grip strength from baseline to the end of treatment in both groups.

During treatment, an undesired complication, oral mucositis was recorded in 91.5% of 48 patients with available data. The effect of compliance status of the patient on the degree of mucositis (light: first degree and second degree; heavy: third degree and fourth degree) was shown in Table 4.

More than one-third (40.7%) of the patients developed second-degree mucositis and one-third of the (30.5%) patients developed third-degree mucositis. Heavy mucositis was significantly less common in compliant than in noncompliant patients (*p* < 0.009).

## 4. Discussion

Malnutrition is frequently seen in HNC patients and it plays a multifactorial role in the progression of the disease. A high rate of tobacco and alcohol use, the lack of a regular lifestyle [13], difficulties in swallowing as a result of the anatomical position of the tumour and the morbidity of the HNC surgery [14], RT dose, sense problems, nausea/vomiting, pain, dry mouth, and having trouble with social eating are significantly associated with malnutrition [2]. In particular, the dysphagia and mucositis due to toxicity of high doses of curative RT were shown to cause a vicious cycle of malnutrition [15]. In a past study malnutrition rates were reported to be 24% before RT, while it was 88% after RT [4]. Weight loss during RT or CRT is a substantial problem in HNC patients and it is accompanied by loss of fat free mass, muscle and organ mass, deterioration in quality of life, more severe treatment-induced toxicity, and a shorter survival [16, 17].

There are beneficial effects of individualized dietary counselling on nutritional status compared to lack of counselling or standard nutritional advice [10]. According to the European Society for Clinical Nutrition and Metabolism [18], the purpose of nutrition counselling and nutritional supplement is to prevent the deterioration of nutrition during treatment and to treat when necessary to reduce treatment-related toxicity and to increase the effectiveness of cancer treatment as well as the quality of life.

In this study, body weight, body composition (muscle mass, fat percentage, fat mass, and fat free mass), and muscle function (hand grip strength) of compliant and noncompliant patients were compared. It was found that 37.5% of patients in our study complied with the recommended nutrition plan, but 62.5% did not, for various reasons. The two groups had similar sociodemographic characteristics. The change in BMI, weight, fat mass, fat free mass, and muscle mass during RT was insignificant in compliant patients whereas, in noncompliant patients, there was a dramatic and significant decrease in these measurements during treatment (*p* < 0.001).

Many clinics place a PEG prophylactically before starting RT in order to prevent weight loss and treatment toxicity [19, 20], while it may regress swallowing function in the long term and may lead to a delay in nutrition via oral route in some patients [21]. Although PEG tube has been suggested to be a more suitable route for enteral nutrition in HNC patients than the NG tube [22], the likelihood of development of complications has also been emphasized [23]. In our cohort, nasogastric feeding tube was applied to three patients and PEG was applied to four patients with full indications.

Nutritional counselling and supplemented oral feeding at the start of treatment have gained great importance. Research has shown that more than 80% of HNC patients develop dysphagia under CRT, and 10% or more lose weight [24]. It has been necessary to suspend treatment because of severe mucositis in 20% of patients [25].

In a previous study in 78 patients with HNC and gastrointestinal cancer by Isenring et al. [26], while minimal weight loss was reported with nutritional supplements, a significant reduction in body weight occurred with standard nutrition. Other studies have shown the benefits of nutritional supplements on fat free mass [10, 27, 28]. In our study fat percentage, fat mass and fat free mass remained unaltered in compliant patients during treatment, whereas they decreased significantly among noncompliant patients during the treatment period.

We assessed nutrition consumption for three consecutive days (two during the week and one at the weekend) in the middle of treatment. Recording of three-day nutrition consumption is important in order to determine energy and protein deficit. We found that the daily energy and protein intake of compliant patients were greater than those of noncompliant patients. The nature of living conditions of patients during RT is important with regard to their food consumption. In our study, compared to those staying in a hotel, patients staying at home or staying with relatives had significantly more sufficient energy intake. This may indicate the higher likelihood of a precise care provided by family or relatives of patients. Both economical issues and desolation of patients receiving RT are important factors in terms of quality of food consumption.

In the present study, no significant muscle loss was observed in compliant patients during RT, but a significant loss was determined in noncompliant patients. Albeit not significant, tendency for similar findings was also noted for muscle function. This may be associated with small sample size and the short-term follow-up period in our study.

In a past study with 75 patients receiving RT for HNC by Ravasco et al. [3], it was shown that merely adding supplement products to a patient's diet was not as effective as dietary counselling. Paccagnella et al. [29] reported that early nutritional intervention in patients with HNC under CRT was associated with an improved treatment tolerance. We observed that mucositis was less severe among patients who complied with nutritional counselling and recommendations than noncompliant patients. The importance of oral nutrition supplement on the toxicity of RT was investigated in a prospective study by Valentini et al. [30]. Radiotherapy-related grade 3 toxicity was found to be greater in those with weight loss, reduced mid-arm circumference, or low serum albumin levels [30]. In our study, no significant weight loss was observed in patients who regularly used the recommended oral nutrition products.

The major strength of this study is, by using BIA prospectively in a special patient population, to present how the nutritional intake may impact body composition such as fat percentage, fat mass, fat free mass, and muscle mass in compliant and noncompliant head and neck cancer patients undergoing RT. Regarding the limitations in our study, we did not monitor nutritional status after completion of RT; thus we did not know whether nutritional status affected the clinician's decision to continue RT. Another limitation is that we considered mucositis being the most important factor on patient's intake and we evaluated only mucositis among the potential factors that may influence good compliance to the dietary treatment.

In conclusion, body composition parameters were better in head and neck cancer patients considered as compliant according to dietary intake than noncompliant ones during radiotherapy period. Further studies addressing cancer symptom management are needed to improve nutritional status and cancer patients should be monitored for malnutrition not only during radiotherapy but also after the completion of treatment.

## Figures and Tables

**Table 1:** Baseline characteristics of patients.

	*n*	%
*Diagnosis *		
Larynx-hypopharynx	29	49.2
Oral cavity	8	13.6
Nasopharynx	5	8.4
Paranasal sinus tumors	5	8.4
Oropharynx	4	6.8
Other	8	13.6
*Stage *		
1	7	11.9
2	15	25.4
3	17	28.8
4	20	33.9
*Type of chemotherapy *		
None	21	35.6
Weekly cisplatin	26	44.1
Neoadjuvant cisplatin-docetaxel followed by 3 weekly cisplatin with concurrent RT	10	16.9
Weekly cetuximab	2	3.4
*Operation type*		
None	31	52.5
Mass excision	9	15.3
Mass excision & cervical dissection	19	32.2

**Table 2:** Percent loss of weight and ONS consumption in overall study population.

	*n*	%
*Weight loss compared to normal weight (%)*		
None	31	52.6
<10%	17	28.8
≥10%	11	18.6
*Type of enteral product recommended (n = 56)*		
Hypercaloric	53	94.6
Diabetic	3	5.4
*Consumption of the recommended ONS (n = 56)*		
Regularly	31	55.4
Not regularly	25	44.6
*Reasons for nonconsumption (n = 28)*		
Loss of appetite, nausea & diarrhoea	14	50.0
Personal reasons (bad taste, not believing the importance of it)	5	17.8
Pain during swallowing	5	17.8
Not stated	4	14.4

**Table 3:** Anthropometric and body composition parameters with respect to compliance.

	Compliant patients (*n* = 18)				Noncompliant patients (*n* = 30)			
	Baseline	Middle of treatment	End of treatment	*p*	Baseline	Middle of treatment	End of treatment	*p*
BMI (kg/m^2^)	25.6 ± 4.2	25.4 ± 4.3	25.3 ± 4.3	0.536	26.9 ± 4.5	26.0 ± 4.4	24.6 ± 4.3^*∗*^	<0.001
Weight	68.7 ± 14.9	69.2 ± 14.9	68.6 ± 14.4	0.666	76.1 ± 14.3	71.3 ± 13.6	67.5 ± 13.0^*∗*^	<0.001
% fat	24.1 ± 10.1	23.8 ± 9.6	23.4 ± 9.2	0.744	25.3 ± 8.2	26.0 ± 8.8	23.6 ± 9.2^*∗*^	<0.001
FM (kg)	17.6 ± 9.4	17.4 ± 8.9	16.8 ± 8.2	0.906	19.2 ± 8.1	18.8 ± 8.1	16.4 ± 7.8^*∗*^	<0.001
FFM (kg)	51.1 ± 8.3	51.7 ± 8.6	51.7 ± 9.1	0.568	54.9 ± 9.9	52.6 ± 9.6	51.2 ± 9.5^*∗*^	<0.001
Muscle mass (kg)	48.6 ± 7.9	49.1 ± 8.8	49.1 ± 8.7	0.568	52.1 ± 9.5	49.9 ± 9.2	48.6 ± 9.1^*∗*^	<0.001

48 patients with three BIA measurements and a food recall were evaluated; Friedman repeated measures; FM: fat mass; FFM: fat free mass

(*∗*) *p* < 0.001 when compared to baseline values.

**Table 4:** Severity of mucositis with respect to compliance.

	Compliant patients		Noncompliant patients	
	*n*	%	*N*	%
Light mucositis (*n* = 30)	16	53.3	14	46.7
Heavy mucositis (*n* = 18)	2	11.1	16	88.9

Chi-square; *p* = 0.009.
